# The United Kingdom National Neonatal Research Database: A validation study

**DOI:** 10.1371/journal.pone.0201815

**Published:** 2018-08-16

**Authors:** Cheryl Battersby, Yevgeniy Statnikov, Shalini Santhakumaran, Daniel Gray, Neena Modi, Kate Costeloe, UK Neonatal Collaborative and Medicines for Neonates Investigator Group

**Affiliations:** 1 Neonatal Data Analysis Unit, Imperial College London, London, United Kingdom; 2 The Society and College of Radiographers, London, United Kingdom; 3 Imperial Clinical Trials Unit, Imperial College London, London, United Kingdom; 4 Barts and the London School of Medicine and Dentistry, London, United Kingdom; Centre Hospitalier Universitaire Vaudois, FRANCE

## Abstract

**Background:**

The National Neonatal Research Database (NNRD) is a rich repository of pre-defined clinical data extracted at regular intervals from point-of-care, clinician-entered electronic patient records on all admissions to National Health Service neonatal units in England, Wales, and Scotland. We describe population coverage for England and assess data completeness and accuracy.

**Methods:**

We determined population coverage of the NNRD in 2008–2014 through comparison with data on live births in England from the Office for National Statistics. We determined the completeness of seven data items on the NNRD. We assessed the accuracy of 44 data items (16 patient characteristics, 17 processes, 11 clinical outcomes) for infants enrolled in the multi-centre randomised controlled trial, Probiotics in Preterm Study (PiPs). We compared NNRD to PiPs data, the gold standard, and calculated discordancy rates using predefined criteria, and sensitivity, specificity and positive predictive values (PPV) of binary outcomes.

**Results:**

The NNRD holds complete population data for England for infants born alive from 25^+0^ to 31^+6^ (completed weeks) of gestation; and 70% and 90% for those born at 23 and 24 weeks respectively. Completeness of patient characteristics was over 90%. Data were linked for 2257 episodes of care received by 1258 of the 1310 babies recruited to PiPs. Discordancy rates were <5% for 13/16 patient characteristics (exceptions: mode of delivery 8.7%; maternal ethnicity 10.2%, Lower layer Super Output Area 16.5%); <5% for 9/16 processes (exceptions: medical treatment for Patent ductus arteriosus 6.1%, high-dependency days 10.2%, central line days 11.2%, type of first milk 22.3%; and during first 14 days, summary of types of milk 13.8%; number of days of antibiotics 9.0%; whether antacid given 5.1%); and <5% for 10/11 clinical outcomes (exception: Bronchopulmonary dysplasia, defined as oxygen dependency at 36 weeks postmenstrual age 3.3%). The specificity of NNRD data was >85% for all outcomes; sensitivity ranged from 50–100%; PPV ranged from 58.8 (95% CI 40.8–75.4%) for porencephalic cyst to 99.7 (95% CI 99.2, 99.9%) for survival to discharge.

**Conclusions:**

The completeness and quality of data held in the NNRD is high, providing assurance in relation to use for multiple purposes, including national audit, health service evaluations, quality improvement, and research.

## Introduction

Accurate data describing interventions and outcomes from well-defined populations are important for monitoring and planning healthcare while also offering opportunities for national and international benchmarking and a platform for clinical research. Worldwide there is a paucity of population based neonatal data [1]. In the United Kingdom healthcare is provided for all through the National Health Service (NHS) funded directly from taxation, and offers opportunity for complete capture of population data.

Neonatal care is a nationally commissioned specialised service delivered through networks of hospitals. The implementation of routine electronic data capture across all networks provides a unique opportunity to acquire population based data without additional data collection systems. Data on newborn infants receiving hospital care whether it be on the neonatal unit, postnatal or transitional care ward are captured on electronic patient records (EPR) held on a web-based platform, BadgerNet, managed by an approved NHS supplier, Clevermed Ltd (Level 6, Edinburgh Quay, 133 Fountainbridge, Edinburgh, EH3 9QG, www.clevermed.com). An extract of over 400 items for each baby forms The Neonatal Dataset (NDS) [2] approved in 2013 as a national NHS Information Standard by the NHS Information Standards Board (now NHS Digital) (ISB1595 version 1.0; now Standardisation Committee for Care Information (SCCI) 1595) [3]. An increasing number of hospitals currently including all of those in England, Wales and Scotland are members of the UK Neonatal Collaborative (UKNC) and the necessary regulatory approvals are in place for the data from each of those hospitals to be transferred quarterly from the BadgerNet platform to the Neonatal Data Analysis Unit (NDAU), an independent research unit of Imperial College London set up in 2007. The NDAU has approvals to use these data to create the National Neonatal Research Database (NNRD). In 2012, the UK Neonatal Collaborative (UKNC) was formed, consisting of all NHS neonatal units contributing data to the NNRD. This database, now includes details of 100,000 infants admitted to neonatal care each year; it has provided data for a wide range of NHS reports and research studies published in Peer reviewed journals [4–8].

Neonatal databases have been established in many countries; the NNRD differs from most by being compiled from EPR with no extra data collection. It is one of the largest clinical databases and holds the largest range of patient characteristics [1]. Data completeness and accuracy are also important considerations, yet formal quality assurance of databases is rarely reported and probably rarely undertaken [9]. In 2010, funding was secured by NDAU from the National Institute of Health Research (NIHR) Medicines for Neonates programme to explore the potential of the NNRD to facilitate research. In this study, we aimed to evaluate the population coverage and data quality of the NNRD data for English hospitals.

## Ethics approval

The National Neonatal Research Database has Research Ethics Approval (London Queen Square Research Ethics Committee Reference number 16/LO/1930).

## Methods

We compared data held in the NNRD with independently collected data from the Office for National Statistics [10] and the Probiotics in Preterm babies Study (PiPS) [11]. The latter was a multi-centre, double blind, placebo-controlled, randomised trial funded by the Health Technology Assessment programme of the UK National Institute for Health Research. Twenty four centres in south-East England recruited patients between July 2010 and July 2013. PiPS trial data were collected using conventional paper Clinical Record Forms (CRF), subjected to a standard series of range, logic and missing data checks and double entered onto a dedicated trial database fulfilling standards of ICH-GCP at the Clinical Trials Unit at the National Perinatal Epidemiology Unit, University of Oxford.

### Data flows

EPR records are held by Clevermed Ltd and stored on a secure NHS server from which individual neonatal units access their data. The NDAU obtained approvals from the Caldicott Guardians of the NHS Trust of each contributing neonatal unit, to receive a predefined data extract (the Neonatal Data Set) from the EPR of each infant admission. Clevermed Ltd transmits these data to the NDAU, where the NNRD is formed. Data are ‘cleaned’ by applying completeness, logic and range checks.

Neonatal services are arranged so that babies move between hospitals according to their clinical need, thus the in-patient period between birth and the first discharge home (or death) may include several episodes in different hospitals. For each infant, to create the NNRD, a single record is compiled by linking the episodes of care across different neonatal units using a unique identifier created by Clevermed, the BadgerID.

**Fig 1:**
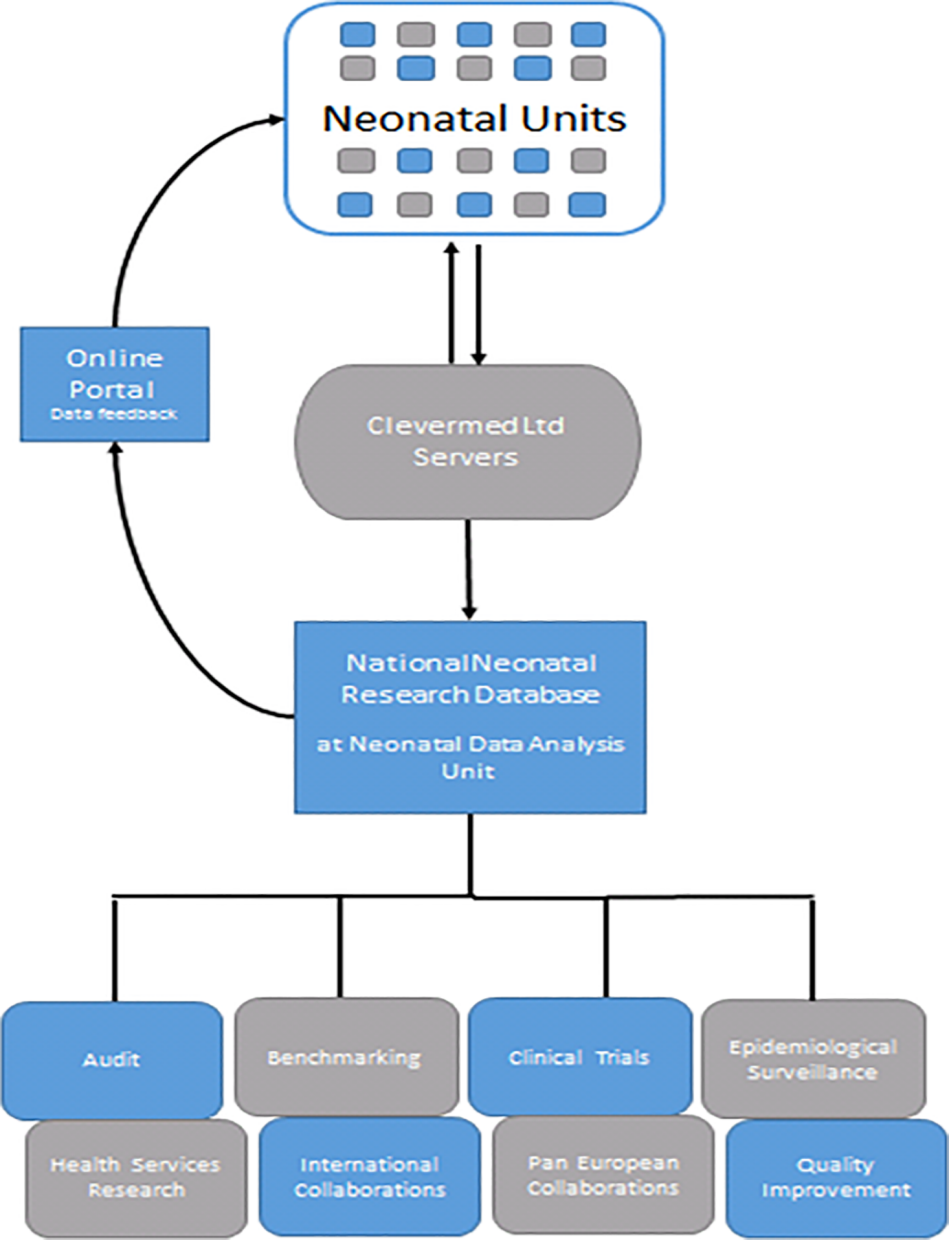
Data flows and outputs from the NNRD.

The NNRD is held on the NHS servers of Chelsea & Westminster NHS Foundation Trust and updated quarterly using MS SQL and SAS programming to include updated patient records from the previous time period. To-date, the NNRD contains data from the year 2006 on over 800,000 infants admitted to NHS neonatal units, and over 10 million care-days. Fig 1 illustrates the data flows and examples of outputs from the NNRD.

### The neonatal dataset

Infants are identified by their unique BadgerID; no patient identifiers (NHS numbers or names) are stored in the NNRD. Age in minutes from birth, and month and year of birth are stored instead of exact dates. An episode of care is defined as a continuous admission in the same neonatal unit. An infant can have multiple episodes of care e.g. if an infant was transferred from hospital A to hospital B, there are two episodes and back to hospital A would be three.

There are three different types of data: demographic details (e.g date and place of birth, birth weight) entered only once for all infants; episodic items (e.g. blood culture, clinical outcomes and diagnoses) which may be entered during each episode of care; and ‘daily’ items that include level of care (special/high dependency/intensive), which is categorised from raw data by embedded programming following data entry, and clinical interventions (e.g. respiratory support, type of feeds, surgical procedures, high cost drugs). Daily location and whether the infant’s mother is resident and providing care are items, required to distinguish between infants cared for on a neonatal unit, postnatal ward or transitional care ward; these data are required for the categorisation of level of care. Diagnoses include fixed choice and some free-text items. Each data item is clearly defined in an accompanying meta-data set, and mapped to existing national standards and ICD codes; conversion to the international medical nomenclature, Snomed CT terminology, is underway [12]. The NDAU is the data guardian; the data controller is Chelsea & Westminster Hospital NHS Foundation Trust. The NDS was approved after data harmonisation was undertaken across NHS datasets assisted by the NHS Data Dictionary team. This included a public consultation to obtain views on included data items; a process undertaken annually to revise the NDS to reflect current practice.

### Population coverage and data quality

We determined the proportion of neonatal units in England contributing data to the NNRD, and the proportion of infants born in England with an NNRD record (by gestational week) in 2008–2014. We obtained denominator figures for the annual number of neonatal units in England and live births from the National Neonatal Audit Programme [13] and Office for National Statistics, respectively [10]. To examine NNRD data completeness, we calculated the percentage of missing data for seven items applicable to all infants (gestational age (GA), sex, birth-weight, antenatal steroids, mode of delivery, multiple birth and survival to discharge from neonatal care). In addition, for antenatal steroids and mode of delivery, we performed a subgroup analysis to determine whether completeness was higher among infants born <32 weeks GA, compared to all GA.

To assess data accuracy at patient level, we performed data linkage between the NNRD and PiPs trial database and compared the agreement between 44 pre-specified items present on both databases (16 patient characteristics, 17 processes, 11 clinical outcomes).

Levels of agreement with criteria for minor and major discordancy were predefined for all 44 items by two of the authors (CB & KC) (S1–S4 Tables). For instance, for a binary item such as whether or not an infant had surgery for a PDA, any difference was considered as a major discordancy whereas for an item such as number of days that a central venous line had been in place a tolerance of +/- 2 days was deemed acceptable, +/- 3–4 days as a minor and +/- 5 or more days as a major discordancy. The 16 patient characteristics were expected date of delivery (EDD), GA, month of birth, year of birth, birth-weight, sex, five minute APGAR score, born in this hospital, singleton or multiple birth, birth order, maternal year of birth, maternal ethnicity, any antenatal corticosteroids given, caesarean or vaginal delivery, instrumental delivery and maternal lower layer super output area (LSOA) (derived from maternal postcode) [14]. In England, the smallest geographical area of practical use, that is, the level at which most national datasets are collected, is the LSOA [15]. These areas are revised after each decennial census to ensure that they contain around 1500 inhabitants. LSOA can be linked to the Index of Multiple Deprivations (IMD) 2010, which reports continuous scores for seven domains of deprivation, for each LSOA in England and Wales [16]. The 17 processes were intensive care days, high dependency care days, central venous line days, length of stay, transfer to another hospital, discharge month, discharge year, surgery for patent ductus arteriosus (PDA), medical treatment for PDA, retinopathy of prematurity (ROP) treatment by laser or cryotherapy, day of first milk, type of first milk, summary of types of milk in the first 14 days, any antibiotics given and number of days of antibiotics in the first 14 days, any antacid given and number of days of antacid given in the first 14 days. The 11 clinical outcomes were worst stage of ROP in any eye, bronchopulmonary dysplasia (defined as supplementary oxygen at 36 weeks postmenstrual age (PMA)), mechanical respiratory support at 36 weeks PMA, any diagnosis of perforated necrotising enterocolitis, any gastrointestinal perforation, any abdominal surgery for NEC, haemorrhagic parenchymal infarct, hydrocephalus, periventricular leucomalacia, porencephalic cyst, and survival to discharge from neonatal care. Analyses were conducted at the level of the episode in each hospital and at infant-level for each infant’s total hospitalisation. The PiPS trial captured EDD, from which GA was calculated, and feeding data for the first 14 postnatal days. Therefore GA discordancy was only calculated for infants with EDD in both databases; ‘first feed’ discordancy was only calculated for infants fed within the first 14 days with no missing data prior to the first reported feed in the NNRD.

### Statistical methods

**Fig 2:**
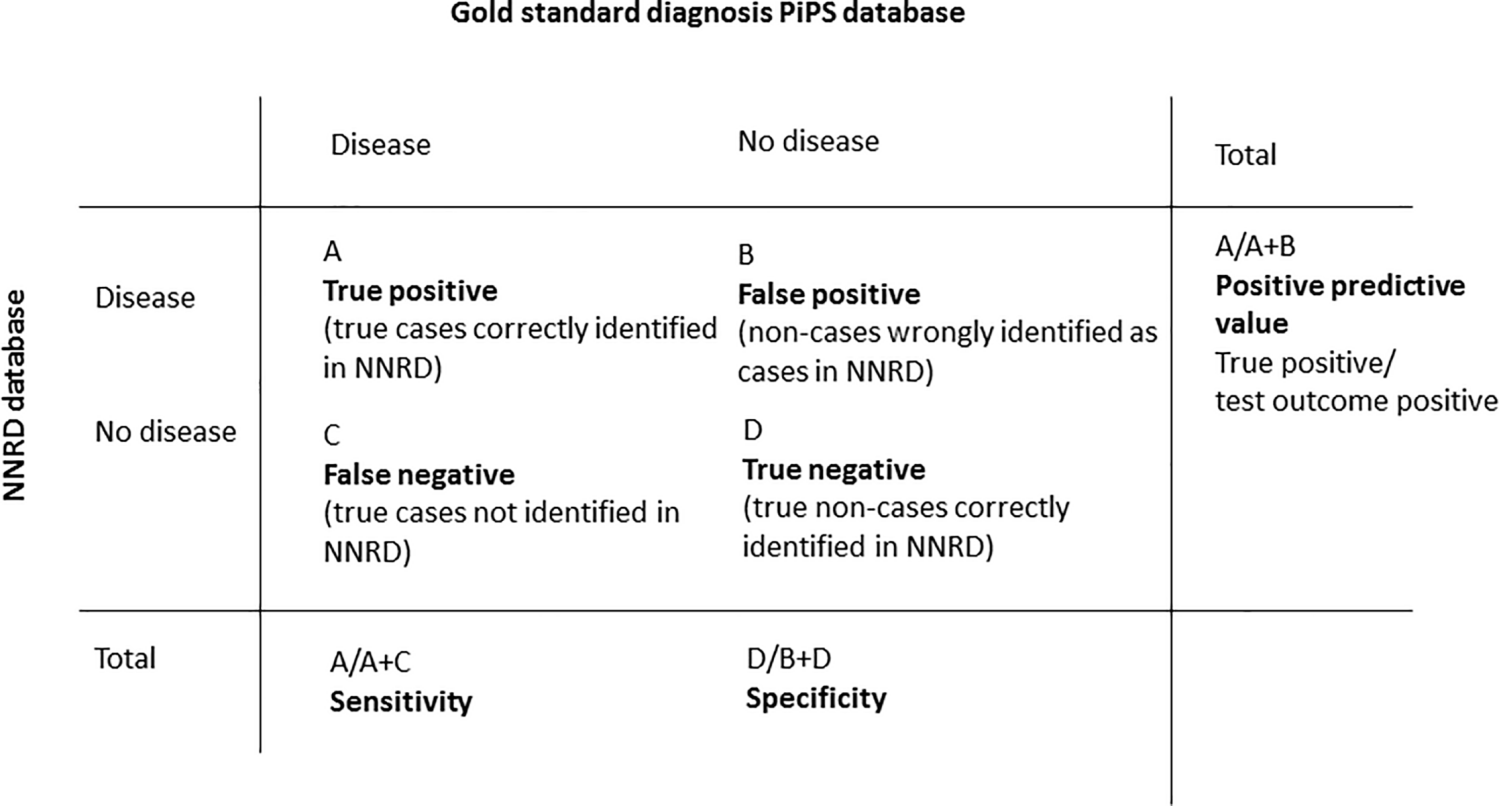
Sensitivity, specificity and positive predictive value of data held on NNRD.

We calculated the percentage of missing data for each item in the PiPs database and the NNRD and minor and major discordances using the predefined criteria. To explore variation of completeness of data by centre we presented the proportion of missing data for incomplete variables using centre-specific box-plots. Infants with missing data were excluded from comparisons. For continuous items, we calculated mean and median differences and 95% limits of agreement for the differences. For binary items, we calculated the percentage of infants for whom the NNRD and PiPS trial data differed with the 95% confidence interval. For items with discordancy rates of less than 5%, we used the Poisson approximation to the Binomial to calculate confidence intervals; otherwise we used the Agresti and Coull method for Binomial confidence intervals as this method has better coverage properties [17]. In addition, for binary processes and outcomes, we calculated sensitivity and specificity, treating PiPs data as the gold standard. We report the prevalence of outcomes in both databases. Sensitivity = number of infants with the disease that are correctly identified by the NNRD/number of infants with the disease identified in the PiPS data. Specificity = the number of infants without the disease correctly identified by the NNRD/number of infants without the disease. PPV = number of infants with the disease that are correctly identified by the NNRD /number of infants (correctly or incorrectly) identified by the NNRD as having the disease. NPV = number of infants without the disease correctly identified by the NNRD/ number of infants (correctly or incorrectly) identified by the NNRD as not having the disease (Fig 2). Analyses were performed using computer codes in SAS version 9.3 and STATA version 11.

#### Regulatory approvals

National Research Ethics Service approval was granted in 2010 to establish the NNRD (10/H0803/151). Caldicott Guardian and Lead Neonatal Clinician approval from every NHS Trust are also held. A Parent Information Leaflet offers parents the opportunity to opt-out, although to date this has not occurred. The Research Ethics Committee advised that the NNRD PiPS data comparison was a data quality assurance study and did not require research ethics approval; a data sharing agreement for this study was agreed between the National Perinatal Epidemiology Unit where the PiPs database was held, and NDAU [11].

## Results

### NNRD population coverage and data completeness

**Fig 3:**
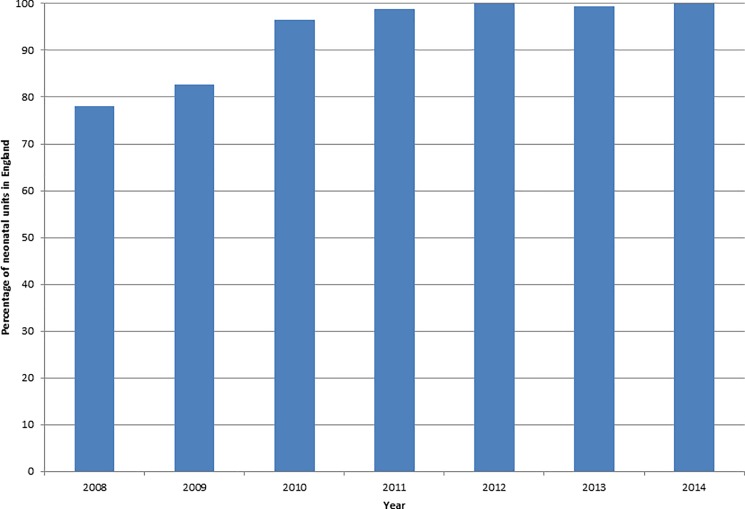
Percentage of neonatal units in England contributing data to the NNRD 2008–2014.

**Fig 4:**
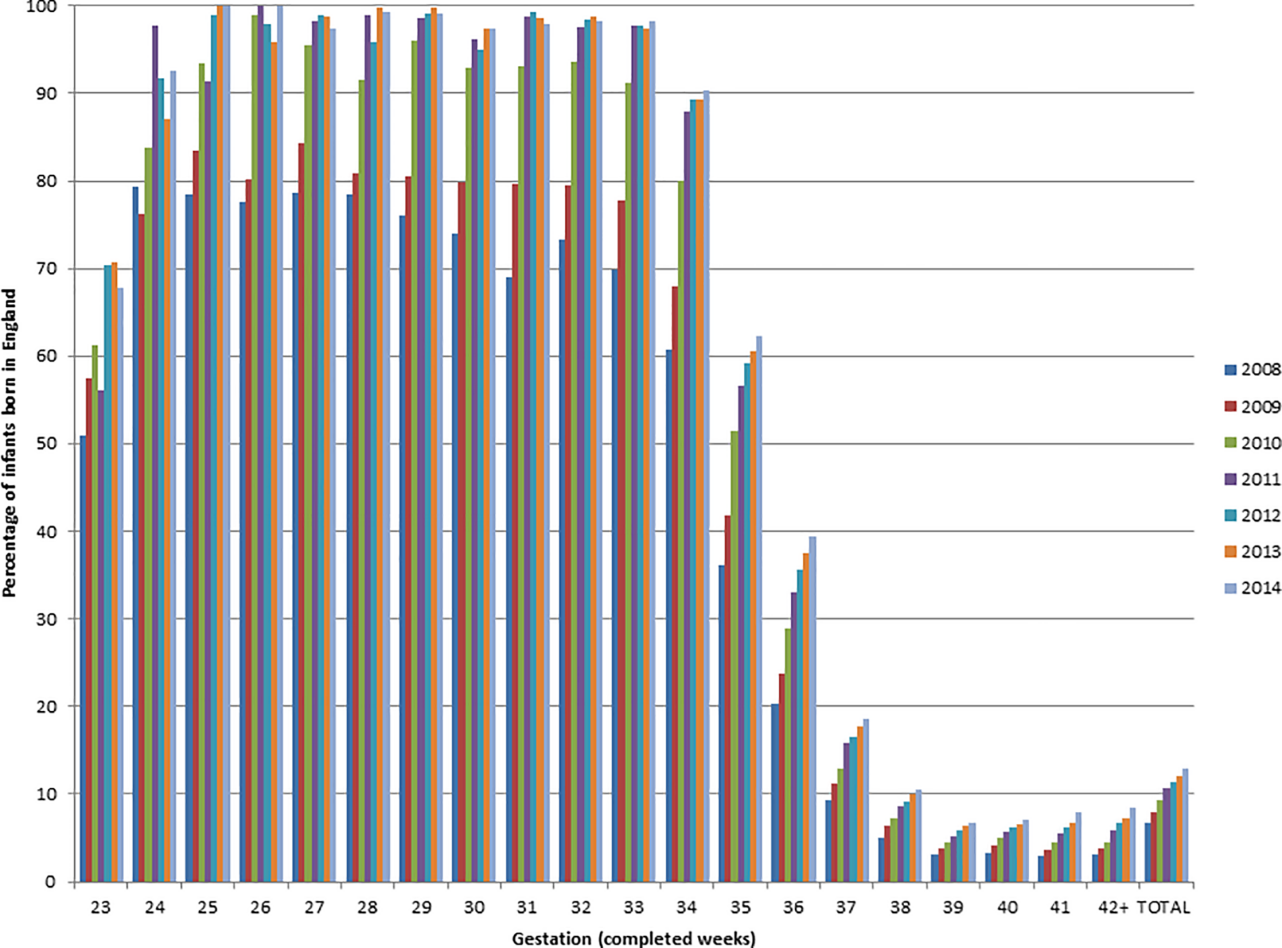
Bar chart showing percentage of infants born in England with an NNRD record.

The proportion of neonatal units in England contributing to the NNRD rose from 78% in 2008 to 100% (163) in 2012 (Fig 3). Closures and mergers resulted in fluctuations in the number of neonatal units over the years. The percentage of live births with an NNRD record increased over the years for all gestational ages, and has been fairly constant since 2012 (Fig 4). Between 2012 and 2014, almost 100% of infants born in England at a GA of 25–31 weeks had an NNRD record; the figures for live born infants at 23w and 24w GA were lower at 70% and over 90%, respectively. The percentage of infants with an NNRD record diminish with increasing GA; 98% for infants 32-33w; 90% 34w; 60% 35w, 40% 36w and 20% 37w. However, over time there has been an increase in the proportion of more mature live births born ≥32 weeks GA with a NNRD record (Fig 4).

Table 1 shows the completeness of seven data items for 568,143 infants admitted over 2008–2015. At national level, well completed data items (<1% missing) in the NNRD include GA, sex, multiple birth and birth-weight. Compared to all GA, the subgroup of infants born <32w GA had less missing data for antenatal corticosteroids (4% <32w; 6.7% all GA), and mode of delivery s (6.7% <32w GA; 20.4% all GA).

**Table 1:** Percentage of infants with an NNRD record with complete data by year of birth.

	Percentage of infants with complete data by year of birth, total births = 568,143							
	2008	2009	2010	2011	2012	2013	2014	2015
Total births with an NNRD record	n = 44620	n = 52908	n = 63129	n = 73131	n = 79136	n = 80409	n = 85064	n = 89746
**GA (completed weeks)**	100.0	100.0	100.0	100.0	100.0	100.0	100.0	100.0
**Sex**	98.9	98.9	100.0	100.0	99.8	100.0	100.0	100.0
**Birth-weight (g)**	98.9	98.9	100.0	99.9	100.0	100.0	100.0	100.0
**Antenatal corticosteroids** All GA	89.5	89.7	89.8	89.8	90.2	91.5	94.6	93.3
Subgroup (only <32w GA)	91.8	93.1	93.2	92.6	95.2	97.1	98.0	96.0
**Mode of delivery** All GA	79.6	79.9	80.5	80.3	79.9	80.7	81.0	79.7
Subgroup (only <32w GA)	84.3	89.0	91.4	92.3	92.2	92.8	93.0	93.3
**Multiple birth**	98.6	98.9	99.9	99.9	99.9	100.0	100.0	99.8
**Final discharge**	96.0	96.5	97.3	99.9	99.6	99.6	99.9	97.9
**Complete data for all 7 variables (all gestation)**	71.1	72.1	73.7	74.4	74.5	77.6	79.3	75.8
**Complete data for all 7 variables (<32 weeks only)**	73.7	78.4	83.1	85.5	87.8	90.3	91.3	84.0

### Assessment of data accuracy

**Fig 5:**
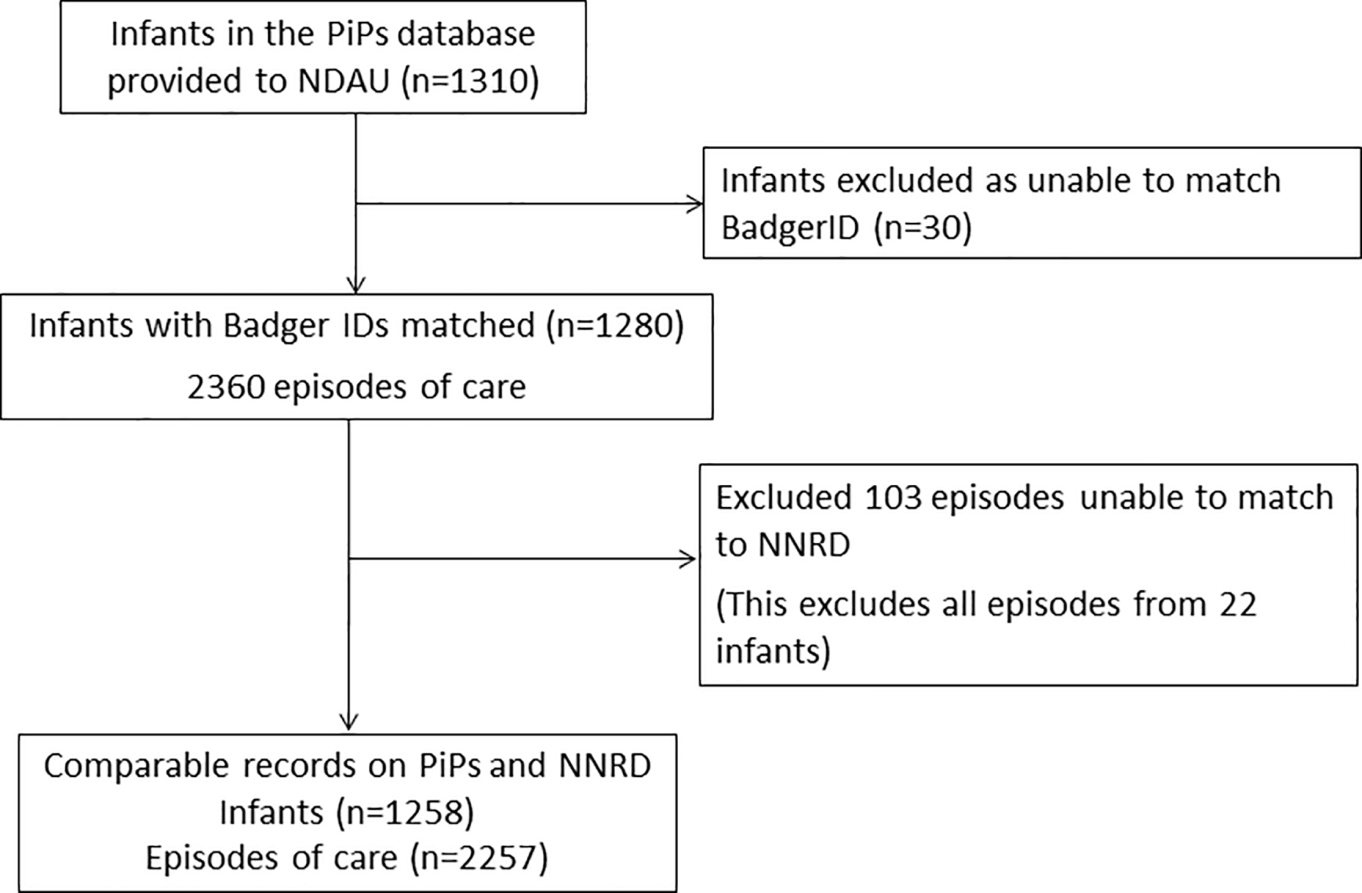
Records from PiPs Clinical Record Forms (CRF) and the NNRD.

Data for 1,310 infants recruited into the PiPs trial recruited in the South East of England over 37 months from July 2010 were available for analysis. Clevermed was able to provide Badger ID for 1,280 (98%) infants. We further excluded 22 infants who had episodes missing from the NNRD database because they occurred in a paediatric ward which does not use BadgerNet, inaccuracies in admission and discharge dates, or inconsistencies in the names of hospitals and NHS Trusts (which were stored as free text on the PiPs database compared to drop down menu on BadgerNet). In total, we excluded 103 episodes of care and 52 infants, leaving a final dataset of 2257 episodes of care from 1258 infants for comparison (Fig 5). Infants with missing data are excluded from calculations of discordancy.

**Fig 6:**
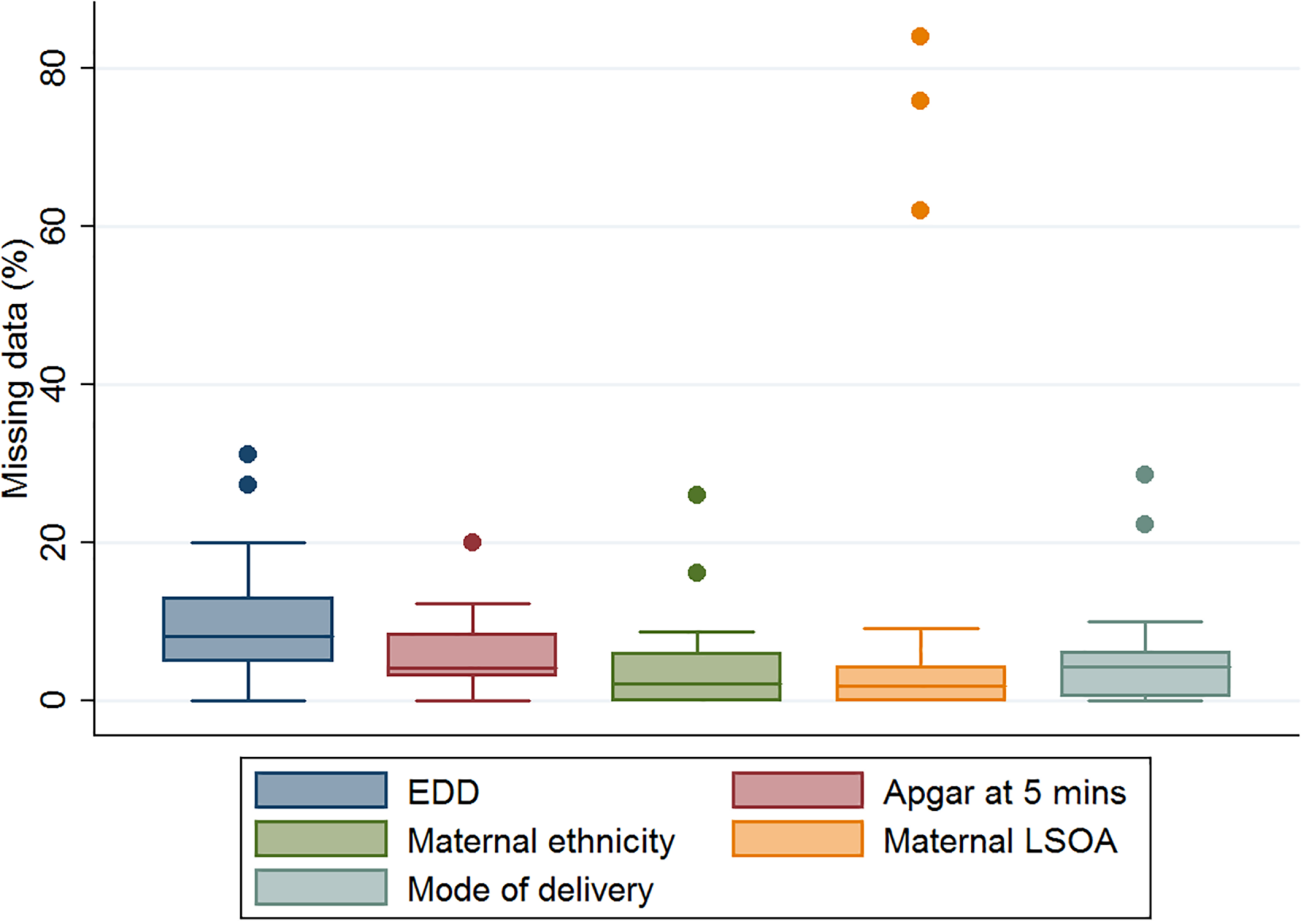
Box plot to show unit variation in data completeness across 24 neonatal units for five variables.

For baseline characteristics, proportion of missing data was higher on the NNRD compared to PiPs database, and >4% missing for EDD, Apgar at 5 minutes, maternal ethnicity, maternal LSOA, and mode of delivery (Table 2). Box-plots show variation in data completeness across 24 PiPS recruiting units for these five variables (Fig 6).

**Table 2:** PiPs vs NNRD: Comparison of baseline infant and maternal characteristics.

		Missing data		Any discordancy^1^		Major discordancy^1^	
Characteristic	No. of comparable infants	PiPs n (%)	NNRD n (%)	Rate (%)	95% CI (%)	Rate (%)	95% CI (%)
Expected date of delivery (EDD)	1142	0	116 (9.2)	9.9	8.3–11.8	4.1	3.0–5.5
GA	1142	0	1	20.4	18.2–22.7	3.0	2.1–4.1
Month of birth	1257	0	1 (0.08)			0	0
Year of birth	1257	0	1 (0.08)			0	0
Birth-weight	1257	0	1 (0.08)	1.7	1.0–2.6	0.9	0.4–1.6
Sex	1256	0	2 (0.16)			0.2	0.02–0.6
Apgar score at 5 minutes	1192	33 (2.62)	63 (5.01)	2.6	1.8–3.7	0.8	0.4–1.5
Born in this hospital	1257	1 (0.08)	0			1.5	0.9–2.4
Singleton or multiple	1257	0	1 (0.08)			1.1	0.6–1.9
Birth order	1257	0	1 (0.08)			0.6	0.3–1.3
Maternal year of birth	1255	0	3 (0.24)			1.4	0.9–2.3
Maternal ethnicity (NHS categories)	1185	10 (0.79)	64 (5.09)			10.2	8.6–12.1
Maternal LSOA	1090	24 (1.91)	151 (12.08)			16.5	14.4–18.8
Any antenatal corticosteroids given	1243	9 (0.7)	6 (0.58)			2.4	1.76–3.4
Caesarean or vaginal delivery	1201	1 (0.08)	56 (4.5)			8.7	7.2–10.4
Instrumental delivery	1248	9 (0.72)	1 (0.08)			1.1	0.6–1.9

^1^ Infants with missing data are excluded from calculations of discordancy

Shaded rows indicates any discordancy is classified as major discordancy

There was no major discordancy for month and year of birth; <1% discordancy for birth weight, sex, APGAR Score at 5 minutes, birth order; 1–3% discordancy for whether born in this hospital, singleton/multiple, maternal year of birth, antenatal steroids and instrumental delivery.

For continuous outcomes, major discordancy (difference of ≥7days) was low for EDD and GA, at 4.1% (95% CI 3.0–5.5%) and 3% (95% CI 2.1–4.1%), respectively and highest for maternal ethnicity (10.2%, 95% CI 8.6–12.1%) and maternal Lower Super Output Area (16.5%, 95% CI 14.4–18.4%) (Table 2).

For processes/interventions, compared at episodic or infant-level, major discordances (difference of ≥5 days), were highest for days of high dependency care and central venous lines at 10.2 (95% CI 9.0–11.5%) and 11.2% (95% CI 10.0–12.6%), respectively (Table 3). Discordancy for medical treatment of a PDA was 6.0%. For all other items (intensive care days, length of stay, transfer, discharge month and year, surgery for PDA, ROP treatment by laser or cryotherapy), discordancies were less than 5%.

**Table 3:** PiPs vs NNRD: Comparison of processes and interventions by episode or infant-level.

		Any discordancy		Major discordancy	
Process/intervention	No. of comparable records	Rate (%)	95% CI (%)	Rate (%)	95% CI (%)
**Comparison by episode**					
Intensive care days	2257	8.5	7.4–9.7	3.9	3.1–4.8
High dependency care days	2257	14.2	12.8–15.7	10.2	9.0–11.5
Central venous line	2257	20.5	18.9–22.2	11.2	10.0–12.6
Length of stay	2257	4.0	3.2–4.9	3.3	2.6–4.2
Transfer to another hospital	2257			2.2	1.6–2.9
Discharge month	2257			2.3	1.8–3.1
Discharge year	2257			0.5	0.3–0.9
**Comparison at infant-level**					
Surgery for PDA	1258			2.0	1.3–2.9
Medical treatment of PDA with ibuprofen or indomethacin	1258			6.1	4.9–7.6
ROP treatment by laser or cryotherapy	1258			1.6	1.0–2.5

For processes in the first 14 days, any antibiotics given had a low discordancy (0.6%, 95% CI 0.2–1.1%) but the number of days given had a major discordancy (>2 days difference) of 9.0% (95% CI 7.6–10.8%) (Table 4). Discordancy for any use of antacid was 5.1% (95% CI 4.0–6.4%) and for number of days given, 4.8% (95% CI 3.7–6.2%). There was high agreement for day of first milk feed, with 2.8% major discordancy (≥2 day difference). There was high discordancy for type of milk given on first day of milk feed (22.3%, 95% CI 19.6–25.1%) and the summary of different milks given over the first 14 days (13.8%, 95% CI 12.0–15.8%).

**Table 4:** PiPs vs NNRD: Comparison of feeds and medications in first 14 postnatal days.

		Any discordancy		Major discordancy	
Process/ intervention	No. of comparable records	Rate (%)	95% CI (%)	Rate (%)	95% CI (%)
Day of first milk	880*	6.7	5.2–8.6	2.8	1.8–4.2
Type(s) of first milk feed	880*			22.3	19.6–25.1
Summary of types of milk in first 14 days (whether given any of: MBM, HDM, formula)	1253^§^			13.8	12.0–15.8
Whether any antibiotics given in first 14 days	1258			0.6	0.2–1.1
Number of days of antibiotics	1258	21.4	19.2–23.7	9.0	7.6–10.8
Whether any antacid was given	1258			5.1	4.0–6.4
Number of days antacid given	1258	6.8	5.6–8.4	4.8	3.7–6.2

*Excluded 35 infants whom were not reported to be fed within the 14 days and 343 infants who had missing data prior to their first reported feed on the NNRD

^§^Excluded 5 infants on the NNRD whose feeding data were missing for the entire 14 days

For all outcomes, discordancy was below 10% except use of oxygen at 36 weeks post-menstrual age which had a discordancy rate of 13.3% (95% CI 11.2–15.8%) (Table 5). Lowest discordancy was for survival to discharge from neonatal care (0.2, 95% CI 0.02–0.6%). Discordancy was 1–2% for worst stage ROP in any eye, any gastrointestinal perforation, hydrocephalus, periventricular leukomalacia; and 2–3% for Any diagnosis of perforated NEC, Any abdominal surgery for NEC, haemorrhagic parenchymal infarct, porencephalic cyst.

**Table 5:** PiPs vs NNRD: Comparison of outcomes.

By infant		Major discordancy	
Outcome	No. of comparable records	Rate (%)	95% CI (%)
Worst stage of ROP in any eye	1258	2.0	1.3–2.9
Whether infant was receiving supplementary oxygen at 36w postmenstrual age (BPD)	877	13.3	11.2–15.8
Whether infant was receiving mechanical respiratory support at 36w postmenstrual age	877	9.2	7.4–11.3
Any diagnosis of perforated Necrotising Enterocolitis (NEC)	1258	2.1	1.4–3.1
Any gastrointestinal perforation	1258	1.7	1.1–2.6
Any abdominal surgery for NEC	1258	2.8	1.9–3.9
Haemorrhagic parenchymal infarct	1258	2.5	1.7–3.5
Hydrocephalus	1258	1.4	0.8–2.2
Periventricular leucomalacia	1258	1.7	1.0–2.6
Porencephalic cyst	1258	2.6	1.8–3.7
Survival to discharge from neonatal care	1258	0.2	0.02–0.6

### Sensitivity and specificity

The prevalence of all outcomes using the NNRD was similar to that derived from the PiPs database (Table 6). The sensitivity of NNRD data for identifying survival was 100% and for adverse outcomes was 50–87%. Specificity was over 85% for all outcomes with the majority above 90%. The prevalence of adverse outcomes among infants <32 weeks is low and less than 6% with the exception of BPD, defined as oxygen dependency at 36 weeks PMA and medical treatment for PDA (49.0% and 20.3% respectively). The PPV of all outcomes with the exception of perforated NEC (66.0%; 95% CI 51.2, 78.8) and details of cerebral ultrasound scans, was over 75% (Table 6).

**Table 6:** Sensitivity, specificity and positive predictive values of key processes and outcomes reported on the NNRD as determined by comparison with PiPs data.

Variable	PiPs positives*	PiPs‡ negatives	NNRD positives*	NNRD negatives‡	Prevalence as per PiPs data % (95% CI)	Prevalence as per NNRD data % (95% CI)	Sensitivity % (95% CI)	Specificity % (95% CI)	Positive predictive value % (95% CI)
	**Processes**								
Surgery for PDA	60	1198	49	1209	4.8 (3.7–6.1)	3.9 (3.0–5.3)	70.0 (56.8–81.2)	99.4 (98.8–99.8)	85.7 (72.8–94.1)
Medical treatment of PDA with ibuprofen or indomethacin	256	1002	189	1069	20.3 (18.2–22.7)	15.0(13.2–17.1)	71.9 (65.9–77.3)	99.5 (98.8–99.8)	97.4 (93.9–99.1)
ROP treatment by laser or cryotherapy	41	1217	49	1209	3.3 (2.3–4.4)	3.9 (2.9–5.1)	85.4 (70.8–94.4)	98.8 (98.1–99.4)	71.4 (56.7–83.4)
	**Outcomes**								
Whether infant required supplementary oxygen at 36w postmenstrual age (BPD)	430	447	433	444	49.0 (45.7–52.4)	49.4 (46.0–52.7)	86.7 (83.2–89.8)	86.6 (83.1–89.6)	86.1 (82.5–89.3)
Whether infant required mechanical respiratory support at 36w PMA	214	663	255	622	24.4 (21.6–27.4)	29.1 (26.1–32.2)	90.7 (85.9–942.2)	90.8 (88.3–92.9)	76.1 (70.4–81.2)
Any diagnosis of perforated Necrotising Enterocolitis (NEC)	43	1215	50	1208	3.4 (2.5–4.6)	4.0 (3.0–5.2)	76.7 (61.4–88.2)	98.6 (97.8–99.2)	66.0 (51.2–78.8)
Any gastrointestinal perforation	55	1203	59	1199	4.4 (3.3–5.7)	4.7 (3.6–6.0)	83.6 (71.2–92.2)	98.9 (98.2–99.4)	78.0 (65.3–87.7)
Any abdominal surgery for NEC	73	1185	60	1198	5.8 (4.6–7.2)	4.8 (3.7–6.1)	67.1 (55.1–77.7)	99.1 (98.3–99.5)	81.7 (69.6–90.5)
Haemorrhagic parenchymal infarct	53	1205	52	1206	4.2 (3.2–5.5)	4.1 (3.2–5.4)	69.8 (55.7–81.7)	98.8 (98.0–99.3)	71.2 (56.9–82.9)
Hydrocephalus	24	1234	18	1240	1.9 (1.2–2.8)	1.4 (0.9–2.3)	50.0 (29.1–70.9)	99.5 (98.9–99.8)	66.7 (41.0–86.7)
Porencephalic cyst	39	1219	34	1224	3.1 (2.2–4.2)	2.7 (1.9–3.8)	51.3 (34.8–67.6)	98.9 (98.1–99.4)	58.8 (40.7–75.4)
Periventricular leukomalacia	40	1218	31	1227	3.2 (2.3–4.3)	2.5 (1.7–3.5)	62.5 (45.8–77.3)	99.5 (98.9–99.8)	80.6 (62.5–92.5)
Survival to discharge from neonatal care	1159	99	1162	96	92.1 (90.5–93.6)	92.4 (90.8–93.7)	100.0 (99.7–100.0)	97.0 (91.4–99.4)	99.7 (99.2–99.9)

*May not be the same infants in PiPs and NNRD positive groups

‡ May not be the same infants in PiPs and NNRD negative groups

## Discussion

This is the first study to formally evaluate the population coverage and accuracy of data held on the NNRD. Completeness and accuracy are fundamental components of data quality (15) yet worldwide there are few published reports on the accuracy of population health data. We believe such assessments to be essential both to confirm the validity of data that potentially underpin a range of important research and service functions and also to highlight areas where modification of the data collection tools will improve data quality.

The number of neonatal units contributing to the NNRD has steadily increased over the years, including all 163 neonatal units in England since 2012, and units in Wales and Scotland since 2015. National ONS data covers all reported live births in England and Wales including any that die in the delivery room and healthy babies with no involvement with neonatal medical services, neither of these groups is entered on the NNRD. Our data show that the NNRD represents complete population-based data for live-born infants born 25 to 31w GA. The discrepancies at 23 and 24w of gestation (70 and 90% representation on the NNRD) are presumably due to death on labour ward and suggest continuing increase of admission rates at these gestations compared with those reported by the population based EPICure studies which in 2006 reported admissions of live births of 64% at 23 and 86% at 24w of gestation [18]. These changes are likely to be related to improved condition at birth and changing attitudes towards the management of extreme preterm infants. We speculate that the increase over time in percentage of infants ≥ 32w with NNRD records may be due to changes in commissioning and a drive to capture for payment purposes medical care outside the neonatal unit e.g. postnatal or transitional care wards.

For baseline patient characteristics, the completeness of data on the NNRD was generally high with the exception of maternal ethnicity and LSOA (derived from maternal postcode), five minute Apgar score and vaginal/caesarean birth. Linkage of maternal and neonatal datasets to create a seamless perinatal dataset would address these problems and avoid the need for duplication of data entry and the risk of transcription errors.

Discordancy was low for most patient characteristics but for processes/interventions we found a high discordancy for the type of feed given on the first day of feeding and in general discordancy was higher for items involving counting days e.g. days of antibiotic treatment in the first 14 days and days with central venous lines. For infant-level outcome data, major discordancy was low except for whether infants were receiving supplementary oxygen on the day they reached 36 weeks PMA (13.3%).

There are a number of possible reasons why differences between the two data sources were found. The choice of data variables for comparison was constrained by what was available on the two databases and while most items describing baseline characteristics were entered onto both in response the same direct question e.g. ‘What was the birthweight?’ the majority of processes/interventions and outcomes were asked for directly at the end of each episode on the PiPS CRF e.g. ‘In this hospital did the infant have a PDA treated surgically?’ whereas in the EPR data could be entered into and extracted from any of three places on the EPR, daily data, discharge diagnoses or procedures during the stay with no direct questions or check lists requiring negative entries. Absence of positive entry on the NNRD was interpreted to mean that the intervention or outcome did not occur whereas it might simply have been missing. This might lead to under reporting within the NNRD and thereby increase discordancy. This problem could easily be overcome with redesign of some entry screens, or the introduction of check lists on the EPR to be completed at discharge.

One of the great strengths of the EPR system underpinning the NNRD and contributing to its richness, is the acquisition of daily data with details of management including items such as the presence of central venous lines, oxygen use, mechanical respiratory support and details of medications. In practice these are used to compute the infant’s level of care (normal, special care, high dependency, intensive) and form the basis of charging within the NHS with mechanisms to avoid double counting when babies move between hospitals. It was agreed when this study was planned that the data on the PiPS trial database should be taken as the gold standard. Data describing length of stay in intensive/high dependency care etc for the PiPS trial were collected in response to the appropriate question at the end of each episode ‘In this hospital for how many days………’ and it is possible that for these items the NNRD data, derived as they are from the raw daily data, are the more accurate.

The levels of agreement and discordancy limits preset by the authors seemed reasonable at the time. As the study proceeded and the complexity of the data including the matching of episodes of care within the total stay emerged, and on subsequent consideration of the structure of the two databases, we have to conclude that it was unrealistic to hope that data describing varying practice such as what different milks a baby received in any one day would be recorded identically in both systems. The accurate recording of complex data such as these and of medications would be helped by standardisation of the structure of questionnaires across clinical and research databases.

We identified high specificity but low sensitivity for some important outcomes. The fact that the PPV was generally high despite low overall prevalence for key outcomes highlights the potential utility of the NNRD as a large and growing population database. Smaller local or regional databases would be unlikely to have adequate statistical power to detect clinically important signals. Overall findings were similar to that of an assessment of the accuracy of routinely collected hospital discharge data in New South Wales against data from a statewide audit of selected neonatal intensive care (NICU) admissions. They also found that, though under-ascertained, routinely collected hospital discharge data had high PPVs for most validated items but that procedures tended to be more accurately recorded than diagnoses [19].

A key strength of our study is the comparison with data from an independent clinical trial conducted to the standards of ICH-GCP. The lack of such a comparator is a common limitation of other database validation studies [9]. We were able to assess patient-level rather than aggregated data, and were able to calculate sensitivity, specificity and negative predictive value, rather than only the PPV as in previous validation studies of the General Practice Research Database (GPRD) [20]. Validation studies often only report the proportion of cases that were confirmed by medical record review or responses to questionnaires, thereby only providing an estimate of PPV. Further, whilst many validation studies have not been blinded or reported by blinded reviewers, our comparisons were automated using computer codes written without knowledge of the dataset identity. We also defined the minor and major discordancy a priori to mitigate bias.

Our study has number of limitations; the principal being the constraints imposed on the scope of the comparison because of lack of standardisation of data items. Also we were not able to validate all episodes held on the PiPs trial database against the NNRD. Data linkage was considered at two levels: first whether an infant recruited into PiPS appeared on the NNRD, second whether all of the episodes of care reported to PiPS were identified on the NNRD. For 2% of recruits into the PiPS trial no EPR data could be identified. Whether this was because of errors of the date of birth and NHS number on either the PiPS database or the NNRD or whether, which seems unlikely, the infants were never entered onto the EPR, is unclear. A further limitation is that the comparison of PiPS and NNRD data was confined to the hospitals participating in the PiPS trial in the South East of England (24 recruiting and 33 step-down sites) and may not be generalisable throughout the UK.

Despite these limitations we have shown that high quality, complete data can be extracted from the routinely collected electronic record and how with some minor changes to the EPR data collection the accuracy of recording of processes, intervention and outcomes within the NNRD could be improved. As electronic records become widely incorporated into daily care and replace paper records, it is expected that data quality will continue to improve. The creation of a static database such as the NNRD, from real-time electronic data is a cost-effective means to create a national resource, obviating the need for duplicate data capture by busy clinical teams, and supporting multiple outputs. The secondary utilities of EPR are increasingly recognised, with advantages that include minimising data entry errors, and better population coverage. The NNRD is now used for a growing number of purposes by a number of research groups, professional organisations and Government bodies [21]. The successful creation of the NNRD is a testament to the collaborative efforts of the UK neonatal community. The NNRD has the potential to revolutionise the approach to conducting clinical research, and offers a time and cost efficient method for conducting clinical trials and population epidemiological studies.

## Supporting information

S1 Table Items selected for comparison: Baseline characteristics, including details of the data held in each database, with pre-set definitions of limits of agreement, and minor and major discrepancies. (DOCX)

S2 Table Items selected for comparison: Processes of care and interventions, including details of the data held in each database, with pre-set definitions of limits of agreement, and minor and major discrepancies. (DOCX)

S3 Table Items selected for comparison: Processes of care and interventions in the first 14 days, including details of the data held in each database, with pre-set definitions of limits of agreement, and minor and major discrepancies. (DOCX)

S4 Table Items selected for comparison: Outcomes, including details of the data held in each database, with pre-set definitions of limits of agreement, and minor and major discrepancies. (DOCX)

## Abbreviations

BPD: Bronchopulmonary dysplasia
CRF: Clinical Record Form
EPR: Electronic Patient Record
GA: Gestational age
HDM: Human Donor Milk
LSOA: Lower Super Output Area
MBM: Maternal Breast Milk
NDAU: Neonatal Data Analysis Unit
NDS: Neonatal dataset
NEC: Necrotising Enterocolitis
NHS: National Health Service
NNRD: National Neonatal Research Database
ONS: Office of National Statistics
PDA: Patent ductus arteriosus
PiPs: Probiotics in Preterm Study
PMA: Postmenstrual age
PPV: Positive Predictive Value
ROP: Retinopathy of Prematurity
