# Supplementary material for: The United Kingdom National Neonatal Research Database: A validation study
Source: PLoS One. 2018 Aug 16;13(8):e0201815. doi: 10.1371/journal.pone.0201815 (PMC6095506; doi:10.1371/journal.pone.0201815)
Supplement: S1 Table — (DOCX) [file pone.0201815.s001.docx]

S1 Table. Items selected for comparison: baseline characteristics, including details of the data held in each database, with pre-set definitions of limits of agreement, and minor and major discrepancies

| **Item to be compared** | **Data held on PiPs** | **Data held on NNRD** | **Definition of limits of agreement** | **Definition of minor disagreement** | **Definition of major disagreement** |
| --- | --- | --- | --- | --- | --- |
| Expected date of delivery (EDD) | EDD | EDD | up to +/- 2 days | 3 - 6 days | +/- 1 week |
| GA (w and days) | GA is computed from EDD | GA (recorded independently of EDD) | up to +/- 2 days | 3 - 6 days | +/- 1 week |
| Month of birth | Month of birth | Month of birth | no difference | N/A | N/A |
| Year of birth | Year of birth | Year of birth | no difference | N/A | N/A |
| Birth-weight (g) | Infant’s birth-weight (g) | Infant’s birth-weight (g) | 30g | 30-100g | >100g |
| Sex | Infant’s sex (Male /Female/indeterminate) | Infant’s sex(Male /Female/indeterminate) | no difference | N/A | N/A |
| Apgar score at 5 minutes | Apgar score at 5 minutes | Apgar score at 5 minutes | +/-1 | +/-2 | +/-3 or more |
| Born in this hospital | Whether infant was born in this hospital | Place of birth | no difference | N/A | N/A |
| Singleton or multiple birth | Whether infant is a singleton or multiple birth | Whether infant is a singleton or multiple birth | no difference | N/A | N/A |
| Birth order | Birth order of infant | Birth order of infant | no difference | N/A | N/A |
| Maternal year of birth | Maternal date of birth | Maternal birth year | no difference | N/A | N/A |
| Maternal ethnicity | Maternal ethnicity (NHS categories) | Maternal ethnicity (NHS categories) | no difference | N/A | N/A |
| Maternal LSOA at time of infant’s birth | Maternal LSOA derived from postcode | Maternal LSOA derived from postcode | no difference | N/A | N/A |
| Whether any antenatal corticosteroids given | Antenatal corticosteroids and exact timing | Any antenatal corticosteroids given, no detail of timing | no difference | N/A | N/A |
| Mode of delivery- caesarean or vaginal | Mode of delivery | Mode of delivery | no difference | N/A | N/A |
| Whether instrumental delivery | Whether forceps or ventouse used for delivery | Mode of delivery | no difference | N/A | N/A |
