# Supplementary material for: The United Kingdom National Neonatal Research Database: A validation study
Source: PLoS One. 2018 Aug 16;13(8):e0201815. doi: 10.1371/journal.pone.0201815 (PMC6095506; doi:10.1371/journal.pone.0201815)
Supplement: S2 Table — (DOCX) [file pone.0201815.s002.docx]

S2 Table. Items selected for comparison: processes of care and interventions, including details of the data held in each database, with pre-set definitions of limits of agreement, and minor and major discrepancies

| **Item to be compared** | **Data held on PiPs** | **Sources of data held for this item on the NNRD** | **Definition of limits of agreement** | **Definition of minor disagreement** | **Definition of major disagreement** |
| --- | --- | --- | --- | --- | --- |
| Surgery for Patent ductus arteriosus | While in this hospital did the infant receive surgical ligation for patent ductus arteriosus (PDA)? | Daily data- surgery for PDA today  Discharge diagnoses  Procedures during stay | no difference | N/A | Any difference |
| Medical treatment for Patent ductus arteriosus with indomethacin or ibuprofen | While in this hospital, has the infant received medical treatment with indomethacin and /or ibuprofen for PDA | Daily data-treatment for PDA  Daily drugs | no difference | N/A | Any difference |
| Treatment for retinopathy of prematurity with laser or cryotherapy | While in this hospital, has infant had ROP treated with laser/ cryotherapy? | Daily data- treatment  Discharge diagnoses  Procedures during stay? | no difference | N/A | Any difference |
| Central venous line days | While in this hospital what was the total number of days for which the infant had a central venous line (UVC, peripheral long line, Broviac etc.) | Daily data –lines insitu | +/-2 days | 3-4 days | +/- 5 or more days |
| Intensive care days | While in this hospital, what was the total number of days of intensive care days? | Daily data | +/-2 days | 3-4 days | +/- 5 or more days |
| High dependency care days | While in this hospital, what was the total number of /high dependency care days? | Daily data | +/-2 days | 3-4 days | +/- 5 or more days |
| Transfer to another hospital | Whether transferred to another hospital | Discharge details | no difference | N/A | Any difference |
| Discharge month | Discharge month | Discharge details | no difference | N/A | Any difference |
| Discharge year | Discharge year | Discharge details | no difference | N/A | Any difference |
