# Supplementary material for: The United Kingdom National Neonatal Research Database: A validation study
Source: PLoS One. 2018 Aug 16;13(8):e0201815. doi: 10.1371/journal.pone.0201815 (PMC6095506; doi:10.1371/journal.pone.0201815)
Supplement: S3 Table — (DOCX) [file pone.0201815.s003.docx]

S3 Table. Items selected for comparison: processes of care and interventions in the first 14 days, including details of the data held in each database, with pre-set definitions of limits of agreement, and minor and major discrepancies

| **Item to be compared** | **Data held on PiPs** | **Source of data held for this item on the NNRD** | **Definition of limits of agreement** | **Definition of minor disagreement** | **Definition of major disagreement** |
| --- | --- | --- | --- | --- | --- |
| Day of first milk | What day of life was milk commenced? | Daily feeding data | +/-1 day | +/-2 days | >2 days |
| Type(s) of first milk feed | Milk on first day of receiving milk feed | Daily feeding data | no difference | N/A | Any difference |
| Summary of all types of milk in first 14 days | Daily feeding data for first 14 postnatal days | Daily feeding data | no difference | N/A | Any difference |
| Total number of days of antibiotics received during first 14 postnatal days | Names and total days of antibiotics received during the first 14 postnatal days | Daily drugs | +/-1 day | +/-2 days | >2 days |
| Total number of days of antacid received during first 14 postnatal days | Total days of antacid use during the first 14 postnatal days | Daily drugs | +/-1 day | +/-2 days | >2 days |
