# Supplementary material for: The United Kingdom National Neonatal Research Database: A validation study
Source: PLoS One. 2018 Aug 16;13(8):e0201815. doi: 10.1371/journal.pone.0201815 (PMC6095506; doi:10.1371/journal.pone.0201815)
Supplement: S4 Table — (DOCX) [file pone.0201815.s004.docx]

S4 Table. Items selected for comparison: outcomes, including details of the data held in each database, with pre-set definitions of limits of agreement, and minor and major discrepancies

| **Variable to be compared, definitions** | **Data held on PiPs** | **Sources of data held for this item on the NNRD** | **Definition of limits of agreement** | **Definition of minor disagreement** | **Definition of major disagreement** |
| --- | --- | --- | --- | --- | --- |
| Worst stage of retinopathy of prematurity in any eye | Worst stage of ROP in ANY eye?(stage 1-5) | Discharge diagnoses  Ad-hoc forms for each ROP examination | no difference | N/A | Any difference |
| Bronchopulmonary dysplasia (BPD) requiring oxygen at 36w post-menstrual age | If still in hospital at 36w post-menstrual age : date reached 36w PMA and whether receiving supplementary oxygen? | Daily data for oxygen use | no difference | N/A | Any difference |
| Requirement of mechanical respiratory support at 36w post-menstrual age | If still in hospital at 36w post-menstrual age was the infant receiving mechanical respiratory support. | Daily data for respiratory support received | no difference | N/A | Any difference |
| Cranial ultrasound findings | While in this hospital, did the infant have any of the following abnormalities in their cranial ultrasound scan?   - Haemorrhagic parenchymal infarct (HPI) - Hydrocephalus (Ventricular index >4mm above 97th centile*) - Porencephalic cyst - Periventricular leucomalacia (PVL) | Discharge diagnoses  Ad-hoc forms for each cranial ultrasound examination | no difference | N/A | Any difference |
| Survival to discharge from neonatal care | Survival to discharge | Discharge details | no difference | N/A | Any difference |
| Gastrointestinal diagnoses | - Perforated Necrotising Enterocolitis (NEC) - Any abdominal surgery for NEC - Any gastrointestinal perforation | Discharge diagnoses  Ad-hoc form reporting AXR  Daily surgery/ NEC data | no difference | N/A | Any difference |
| Length of stay | What was the total length of stay in neonatal care? | Daily data  Discharge details | +/-1 day | +/- 2 days | +/- 3 or more days |
